# Comparative 5’ IsomiRome Analysis Uncovers Dysregulated 5’ IsomiRs in *Trypanosoma cruzi*-infected Macrophages

**DOI:** 10.34133/csbj.0146

**Published:** 2026-06-24

**Authors:** Ming-Ju Amy Lyu, Munire Maimaiti, Jiameng Hu, Haiyang Hu

**Affiliations:** ^1^ Central Laboratory, Innovation and Incubation Center, Shanghai Pulmonary Hospital, School of Medicine, Tongji University, Shanghai 200433, China.; ^2^ State Key Laboratory of Plant Molecular Genetics, Center of Excellence for Molecular Plant Sciences, Chinese Academy of Sciences, Shanghai 200032, China.; ^3^ State Key Laboratory of Natural Medicines, School of Life Science and Technology, China Pharmaceutical University, Nanjing, China.

## Abstract

*Trypanosoma cruzi* (*T. cruzi*), the etiological agent of Chagas disease, remains a major global health threat with limited therapeutic options. Macrophages are central to host defense against *T. cruzi*, yet their failure to achieve complete parasite clearance contributes to disease progression. Although 5’ isomiRs have emerged as functional regulators of gene expression, their expression alternations in *T. cruzi*-infected macrophages remain largely unknown. Here, we performed comparative analyses of 5’ isomiR expression in *T. cruzi*-infected cells and identified a marked, selective increase in microRNA 5’-end heterogeneity in THP-1-derived macrophages but not in cardiomyocytes or epithelial cells. Comparative 5’ isomiRome analysis revealed 68 differentially expressed 5’ isomiRs in infected macrophages, most of which were specific to *T. cruzi* relative to other pathogens. Among them, 56 were associated with Argonaute proteins, including 3 derived from the miR-1246 precursor. Focusing on miR-1246|+1, a 5’ isomiR generated by a 1-nucleotide downstream shift at the 5’ end of miR-1246, we showed that its overexpression substantially down-regulated target genes involved in nuclear factor-κB (NF-κB) signaling, circadian rhythm, cytokine responses, and cell migration. Notably, miR-1246|+1 broadly suppressed NF-κB family transcription factors and their downstream effector genes, thereby inhibiting proinflammatory M1 macrophage phenotype. Intriguingly, the suppressed NF-κB regulators Nfkb1 and Rela, together with M1 macrophage signature genes Ccl8, Il1a, Il1b, and Tlr2, displayed rhythmic expression in murine peritoneal macrophages. Collectively, these findings reveal cell type- and pathogen-specific reprogramming of the 5’ isomiR landscape in *T. cruzi*-infected macrophages and identify miR-1246|+1 as a potential posttranscriptional regulator of macrophage inflammatory polarization.

## Graphical Abstract

**Figure:**
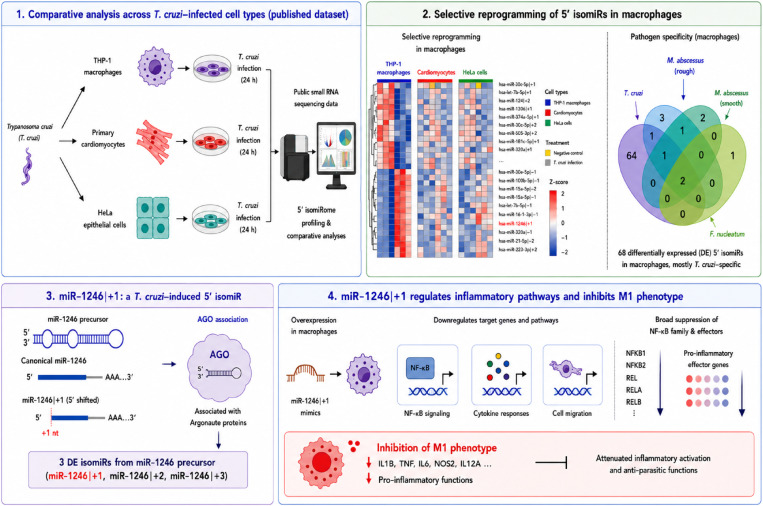

## Introduction

*Trypanosoma cruzi* (*T. cruzi*), the etiologic agent of Chagas disease, remains a major global health threat and a leading cause of infectious cardiomyopathy. Although vector control and screening programs have reduced transmission in some endemic settings, Chagas disease continues to affect millions of people worldwide and is increasingly recognized in nonendemic regions because of population mobility [1–4]. After acute infection, most individuals enter a prolonged indeterminate phase, yet up to one-third subsequently develop clinically relevant cardiac disease, including chronic cardiomyopathy, arrhythmias, thromboembolic complications, and heart failure [2,3,5,6]. Despite longstanding use of trypanocidal agents, therapeutic benefit in established chronic Chagas cardiomyopathy remains limited, underscoring the need to better define host–parasite interactions that drive persistence and pathology [2,7].

Macrophages are among the earliest and most influential host cells engaged during *T. cruzi* infection. They contribute to parasite control through nitric oxide production, inflammatory cytokine release, and coordination of downstream adaptive immune responses [8,9]. In particular, classically activated M1-like macrophages are closely linked to protective antiparasitic activity, as they generate nitric oxide and proinflammatory chemokines and cytokines such as tumor necrosis factor and several interleukins, which restrict intracellular parasite replication and promote resistance to infection [8,9]. However, this protective program is often incomplete, transient, or actively subverted, allowing macrophages to serve not only as effector cells but also as a permissive intracellular niche that supports parasite persistence and fuels chronic inflammatory tissue damage. Thus, understanding how M1-associated macrophage responses are established, maintained, and suppressed during *T. cruzi* infection is central to resolving the balance between host protection and disease progression. A defining feature of *T. cruzi* pathogenesis is its capacity to modulate host immunity. Previous studies have shown that the parasite interferes with inflammatory signaling pathways, including nuclear factor-κB (NF-κB)-associated responses, and exploits host regulatory processes such as efferocytosis to attenuate microbicidal macrophage functions [10–12]. Notably, these mechanisms converge on suppression of M1-like inflammatory activation, thereby weakening macrophage-mediated parasite control and favoring intracellular survival [10–12]. Together, these observations support a model in which parasite persistence is facilitated not only by immune evasion in the classical sense but also by active rewiring of host inflammatory programs. However, the molecular regulators that coordinate this macrophage reprogramming, particularly at the posttranscriptional level, remain incompletely understood.

MicroRNAs (miRNAs) are key posttranscriptional regulators of macrophage activation and inflammatory signaling, shaping the magnitude and duration of NF-κB activity, cytokine production, and polarization states [13,14]. miRNA biogenesis is a tightly controlled multistep process involving Pol II transcription, followed by Drosha- and Dicer-mediated processing to generate mature miRNAs. The functional guide strand is then loaded into Argonaute (AGO) proteins to form the RNA-induced silencing complex (RISC). Target recognition is primarily determined by the seed region at the 5’ end of the miRNA, enabling repression of target mRNAs specifically [15–19]. Beyond canonical miRNAs, individual miRNA loci produce multiple sequence variants known as isomiRs, which substantially expand the functional complexity of the small RNA landscape [20,21]. Among these, 5’ isomiRs are of particular interest because variation at the 5’ terminus alters the seed sequence and can redirect target selection, thereby enabling context-dependent rewiring of regulatory networks [20,21]. Consistent with this, we and others have shown that infection influences not only total miRNA abundance but also the relative expression of distinct isomiR species [21–23]. Nevertheless, whether 5’ isomiRs contribute to macrophage reprogramming during *T. cruzi* infection, especially in relation to suppression of inflammatory M1-like phenotype, has remained largely unexplored.

In this study, we investigated the 5’ isomiR landscape across human cell types in response to *T. cruzi* infection, with a particular focus on macrophages. We found that infection selectively induced increased 5’-end heterogeneity in THP-1 macrophages and identified a set of *T. cruzi* infection-responsive 5’ isomiRs in a cell-type and pathogen-specific manner, many of which showed evidence of AGO association. Analyses of transcriptomic changes and functional enrichment further supported a role for 1 5’ isomiR, miR-1246|+1, in restraining NF-κB signaling, circadian rhythm, inflammatory cytokine responses, and cell migration, thereby limiting proinflammatory M1-like phenotype. Interestingly, several suppressed NF-κB transcription factors and canonical M1 macrophage signature genes displayed rhythmic expression in murine peritoneal macrophages. Collectively, these findings identify 5’ isomiR remodeling as a previously underappreciated layer of host regulatory plasticity during *T. cruzi* infection specifically in macrophage and suggest miR-1246|+1 as a candidate mediator of *T. cruzi*-favored immune modulation.

## Materials and Methods

### Identification and nomenclature of 5’ isomiRs

5’ IsomiRs were identified using our previously established 5’ isomiR identification pipeline [23]. Briefly, raw reads from small RNA sequencing (RNA-seq) data were first trimmed to remove 3’ adaptor sequences using FindAdapt with default parameters [24], and the processed reads were then aligned to the human genome (hg38) using Bowtie with the parameters -v 1 -a --best --strata [25]. To minimize false-positive identification of 5’ isomiRs arising from cross-mapping among miRNA family members [26], only reads with perfect matches were retained for downstream analysis. Mapped reads were subsequently clustered according to their 5’ genomic start positions. 5’ IsomiRs were defined as miRNA isoforms exhibiting 5’-end shifts within ±6 nucleotides relative to the 5’ ends of annotated canonical miRNAs, based on miRBase annotations [27]. This ±6-nt window was selected because more than 99% of reads mapped to miRNA precursors fall within this region relative to the 5’ ends of annotated canonical miRNAs. Each identified 5’ isomiR was named using the corresponding anomiR name from miRBase together with the direction and magnitude of the 5’-end shifts. Specifically, a minus sign (−) indicates that the 5’ end of the isomiR is shifted upstream relative to its cognate anomiR, whereas a plus sign (+) indicates a downstream shift. The number following the sign denotes the number of nucleotides by which the 5’ end is shifted from that of the canonical miRNA. Small RNA-seq data of human THP-1-derived macrophages, primary cardiomyocytes, and HeLa cells (epithelial cells) infected with *T. cruzi* were downloaded from National Center for Biotechnology Information under the accession number PRJNA607998. Small RNA-seq data of THP-1-derived macrophages infected with *Trichophyton rubrum*, *M. abscessus* (rough variant), *M. abscessus* (smooth variant), *M.tuberculosis*, and *F. nucleatum* were downloaded from National Center for Biotechnology Information under the accession numbers GSE149222, GSE72769, GSE184660, and GSE190606, respectively. We then applied the above pipeline to identify 5’ isomiRs in these datasets.

### Quantification and differential expression analysis of 5’ isomiRs

For each identified 5’ isomiR, its expression was estimated using the sum of mapped reads with the 5’ end exactly matching with the corresponding 5’ isomiR. For the multimapping reads, expression levels were normalized by the number of mapped loci. In each pathogen-infected dataset, to identify differentially expressed 5’ isomiRs caused by pathogen infection, the expression of 5’ isomiRs were normalized using the trimmed mean of M-values method across samples, and then the differentially expressed 5’ isomiRs were identified using negative binomial test based on the edgeR package [28] by taking control mimics infected cells as negative control. The primary threshold was false discovery rate (FDR) < 0.05 with an additional fold-change cutoff of 1.5, corresponding to |log2FC| ≥ 0.585. To further assess the robustness of differential expression calls, an independent differential expression analysis was performed using the DESeq2 package [29] based on a negative binomial generalized linear model with median-of-ratios normalization. Consistent differential expression patterns were observed between edgeR and DESeq2 analyses, supporting the reliability of the identified pathogen-responsive 5′ isomiRs.

### Expression pattern analysis across AGO proteins

For the AGO proteins (AGO1, AGO2, and AGO3) that were immunoprecipitated (AGO1-IP, AGO2-IP, and AGO3-IP), small RNA-seq data of THP-1 cells were downloaded from the DDBJ Sequence Read Archive (DRA) under accession number DRA000205. For 68 differentially expressed 5’ isomiRs identified in *T. cruzi*-infected THP-1-derived macrophages, we applied the method in the section “Quantification and differential expression analysis of 5’ isomiRs” to quantify their expression in each of the 3 AGO proteins. The expression patterns of 5’ isoforms across 3 AGO proteins were further analyzed using the R package Mfuzz with default settings [30]. The optimal number of clusters was determined using both hierarchical clustering (hclust) and the elbow method, which consistently supported a 4-cluster solution. Mfuzz clustering was subsequently repeated 500 times with the cluster number set to 4 to ensure the robustness of the identified expression patterns.

### Cell culture

Human monocytic cell line THP-1 (American Type Culture Collection TIB-202) were maintained in RPMI 1640 medium (Gibco, Thermo Fisher Scientific; Cat# 11875-093) supplemented with 10% heat-inactivated fetal bovine serum (Gibco, Cat#A5256701) and 0.05 mM 2-mercaptoethanol (Gibco; Cat# 21985-023) at 37 °C in a humidified atmosphere containing 5% CO₂, according to according to the manufacturer’s instructions. THP-1 cells were grown in suspension and passaged every 2 d to maintain a cell density between 2 × 10^5^ and 1 × 10^6^ cells/ml. Cells in the logarithmic growth phase with viability >95% (as determined by trypan blue exclusion) were used for all experiments.

### PMA-induced differentiation

THP-1 cells were differentiated into macrophage cells by treatment with phorbol 12-myristate 13-acetate (PMA) (Sigma-Aldrich; Cat# P8139). Briefly, cells were seeded at an appropriate density and incubated with 100 nM PMA for 24 h at 37 °C in a humidified atmosphere containing 5% CO₂. Following stimulation, cells were washed with phosphate-buffered saline to remove residual PMA and further cultured in fresh complete medium for an additional 24-h resting period to allow stabilization of the differentiated phenotype prior to subsequent experiments.

### miRNA mimics transfection experiment

Single-stranded miRNA mimics are chemically synthesized RNA molecules that mimic the function of endogenous miRNAs and are commonly used in functional analyses to up-regulate miRNA activity by reproducing their natural function and observing the resulting cellular effects. Single-stranded miRNA mimics of miR-1246|+1 (miR-1246|+1 mimics) and control mimics were synthesized by GenePharma (Shanghai, China). For transfection experiment, PMA-treated THP-1 cells were transfected with miR-1246|+1 mimics or control mimics (60 nM final concentration) using the siRNA-Mate plus transfection reagent (Genepharma Cat#G04026) according to the manufacturer’s protocol. After 24 h, the transfection medium was replaced with fresh complete medium (RPMI 1640). Cells were harvested at 72 h posttransfection for RNA-seq experiments. The transfection experiments were performed in 2 biological replicates, resulting in a total of 4 RNA-seq datasets.

### RNA-seq experiment

RNA-seq experiments were performed as previously described [31–33]. Total RNA was extracted from PMA-treated THP-1 cells transfected with miR-1246|+1 mimics or control mimics using TRIzol reagent (Invitrogen; Cat# 15596018CN), according to the manufacturer’s instructions. RNA quality was assessed using an Agilent 2100 Bioanalyzer, and samples with an RNA integrity number greater than 8.0 were used for subsequent library construction. Poly(A)+ RNA was enriched from total RNA and used for library preparation with the TruSeq RNA Library Preparation Kit v2 (Illumina), following the manufacturer’s protocol. The resulting cDNA libraries were sequenced on an Illumina HiSeq 4000 platform using a paired-end 150-bp (2 × 150 bp) configuration.

### RNA-seq data analysis

RNA-seq data analysis was performed as previously described [31–33]. Briefly, raw RNA-seq reads were processed with Trimmomatic [34] with default parameters to remove low-quality reads and potential adaptor contamination. High-quality reads of at least 75 nucleotides in length were then aligned to the human reference genome (hg38) using HISAT2 [35] with default parameters. Only uniquely mapped reads were used for gene-level quantification via featureCounts [36]. The human genome and gene annotation files were obtained from the Ensembl FTP site (ftp://ftp.ensembl.org/pub). Differentially expressed genes were primarily identified with the edgeR package [28] using negative binomial test after normalization by the trimmed mean of M-values method. DESeq2 package [29] was also used to identify differentially expressed genes based on a negative binomial generalized linear model after normalization by the median-of-ratios method. The primary threshold was FDR < 0.05 with an additional fold-change cutoff of 1.5, corresponding to |log2FC| ≥ 0.585.

### Functional enrichment analysis

Enriched Gene Ontology biological processes were identified using the DAVID Bioinformatics Resources (https://davidbioinformatics.nih.gov/). Significantly enriched processes were determined using a FDR threshold of <5%, with all expressed genes used as the background.

### Protein–protein interaction network analysis

Protein–protein interaction (PPI) information was downloaded from the STRING database [37]. We constructed a PPI network of 321 significantly repressed target genes of miR-1246|+1 and performed clustering analysis using the K-means method. For each cluster, analysis of observed number of edges, expected number of edges, and PPI enrichment *P* value were calculated in the STRING database.

### miRNA target gene prediction

Target genes of miR-1246|+1 were predicted using a combination of sequence-based and expression-based criteria. The seed sequence of miR-1246|+1 was defined as nucleotides 2 to 8 at the 5’ end. Human 3’ untranslated regions of protein-coding genes were scanned for canonical seed-matched sites, including 8mer, 7mer-m8, and 7mer-A1 sites. Only genes expressed in our RNA-seq dataset were retained for downstream analysis. Functionally relevant targets were then identified as those significantly down-regulated following transfections with miR-1246|+1 mimics, using a threshold of FDR < 0.05 and log2 fold change < −0.585.

### Circadian rhythm analysis

Circadian expression of Cluster1 genes was assessed using publicly available time-series data from murine peritoneal CD11b^+^ macrophages [38]. Processed rhythmicity information was obtained from CircaKB (https://cdsic.njau.edu.cn/CircaKB/#/home), a curated knowledgebase integrating and reanalyzing circadian transcriptomic datasets. Genes were considered rhythmically expressed if they met the thresholds of *P* value < 0.05 and amplitude > 1.2-fold. Seven genes from Cluster1 annotated to the Gene Ontology term “circadian rhythm” (Bhlhe41, Arntl2, Egr3, Klf10, Slc6a4, Nampt, and Tnfrsf11a) were evaluated for rhythmic expression patterns. In addition, rhythmic expression of NF-κB transcription factors and M1 macrophage signature genes was examined to assess the intersection of circadian regulation with inflammatory programs.

### Real-time PCR

The quantification of selected gene was conducted by real-time polymerase chain reaction (PCR) using the SYBR Green PCR mix (Applied Biosystems). The obtained values were normalized to the level of glyceraldehyde 3-phosphate dehydrogenase mRNA. The primers used are listed as follows:

*NFKB1*, Forward: AGCAAATAGACGAGCTCCGA

Reverse: TCGGTAAAGCTGAGTTTGCG

*NFKB2*, Forward: GGAGCAAGAGGCCAAAGAAC

Reverse: CCTGCTGTCTTGTCCATTCG

*REL*, Forward: TGTTACCCACTCTGCCTCTG

Reverse: AGTAAGGAGGCAGGACCAAC

*RELA*, Forward: GGGATGGCTTCTATGAGGCT

Reverse: CTGACTGATAGCCTGCTCCA

*RELB*, Forward: TAACAACCTGGGCATCCAGT

Reverse: GATGGTTCTTCAGGGACCCA

## Results

### *T. cruzi* infection drives substantial 5’-end heterogeneity of miRNAs in macrophages

**Fig. 1.**
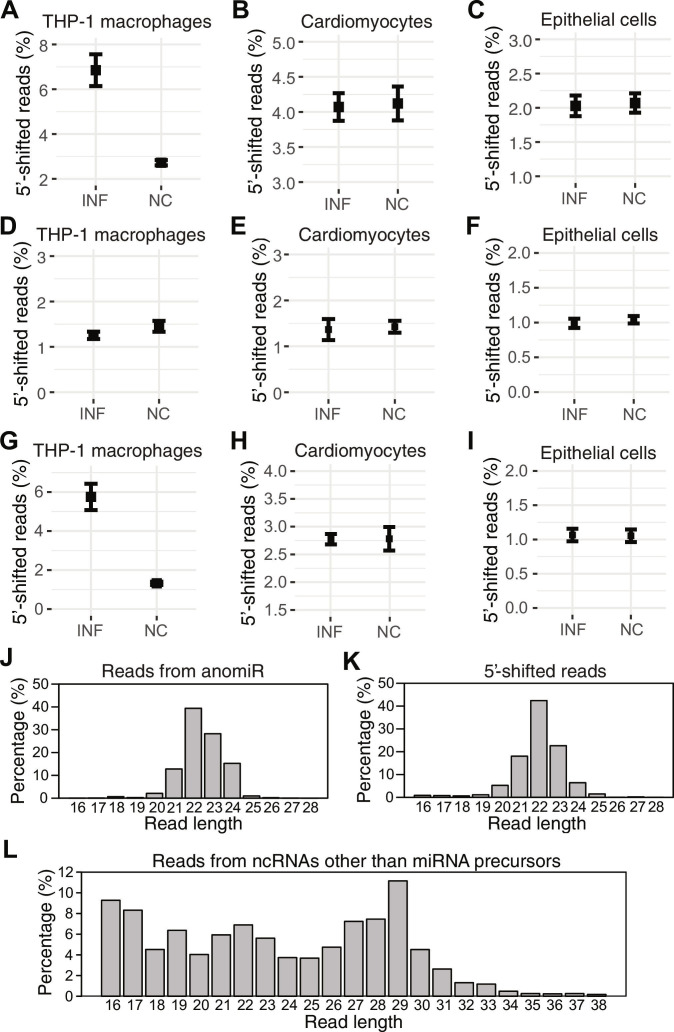
*Trypanosoma cruzi* infection induces substantial 5’-end heterogeneity of microRNAs (miRNAs) in macrophages. (A to C) Proportions of reads exhibiting 5’-end shifts to the upstream or downstream of cognate anomiRs in *Trypanosoma cruzi-*infected THP-1-derived macrophages (A), primary cardiomyocytes (B), and epithelial cells (HeLa cells) (C). (D to F) Proportions of reads exhibiting 5’-end shifts to the upstream of the 5’ end of cognate anomiRs in *Trypanosoma cruzi*-infected THP-1-derived macrophages (D), primary cardiomyocytes (E), and epithelial cells (F). (G to I) Proportions of reads exhibiting 5’-end shifts to the downstream of the 5’ end of cognate anomiRs in *Trypanosoma cruzi*-infected THP-1-derived macrophages (G), primary cardiomyocytes (H), and epithelial cells (I). (J to L) Read distribution of each length for reads derived from anomiRs (J), 5’-shifted reads of anomiRs (K), and noncoding RNAs (ncRNAs) other than miRNA precursors (L) in *Trypanosoma cruzi*-infected THP-1-derived macrophages.

To investigate whether *T. cruzi* infection affects the 5’-end heterogeneity of miRNAs, we quantified the distribution of 5’-shifted reads relative to annotated canonical miRNAs across THP-1-derived macrophages, primary cardiomyocytes, and epithelial cells (HeLa cells) after *T. cruzi* infection (Materials and Methods). In THP-1-derived macrophages, *T. cruzi* infection resulted in a pronounced increase in the proportion of miRNA reads exhibiting 5’-end shifts, whereas only minimal changes were observed in primary cardiomyocytes and epithelial cells (Fig. 1A to C), indicating that *T. cruzi* infection induced a strong and cell type-restricted remodeling of miRNA 5’-end usage. To further dissect the nature of these 5’-end heterogeneities, we next separated 5’-end shifts according to their direction relative to the annotated canonical miRNA 5’ end. Upstream-shifted reads are shown in Fig. 1D to F, whereas downstream-shifted reads are shown in Fig. 1G to I. While both types of shifts were detectable, downstream shifts accounted for the majority of the increase of 5’-end heterogeneity in *T. cruzi*-infected macrophages (Fig. 1D to I), suggesting a directional bias in 5’ isomiR generation. This bias implied that the observed heterogeneity was not random but instead might reflect regulated alterations in miRNA processing or stabilization. To exclude the possibility that these 5’-end heterogeneities arise from nonspecific RNA degradation or contamination from other RNA species, we examined the length distribution of the sequencing reads. Reads corresponding to 5’-shifted reads displayed a canonical peak at approximately 22 nucleotides, closely resembling the distribution of annotated miRNAs, whereas reads derived from other noncoding RNAs exhibited distinct length profiles (Fig. 1J to L). Together, these results demonstrated that *T. cruzi* infection induced a substantial of 5’ isomiR diversity specifically in macrophages.

### *T. cruzi* infection selectively reprograms 5’ isomiR expression in macrophages

**Fig. 2.**
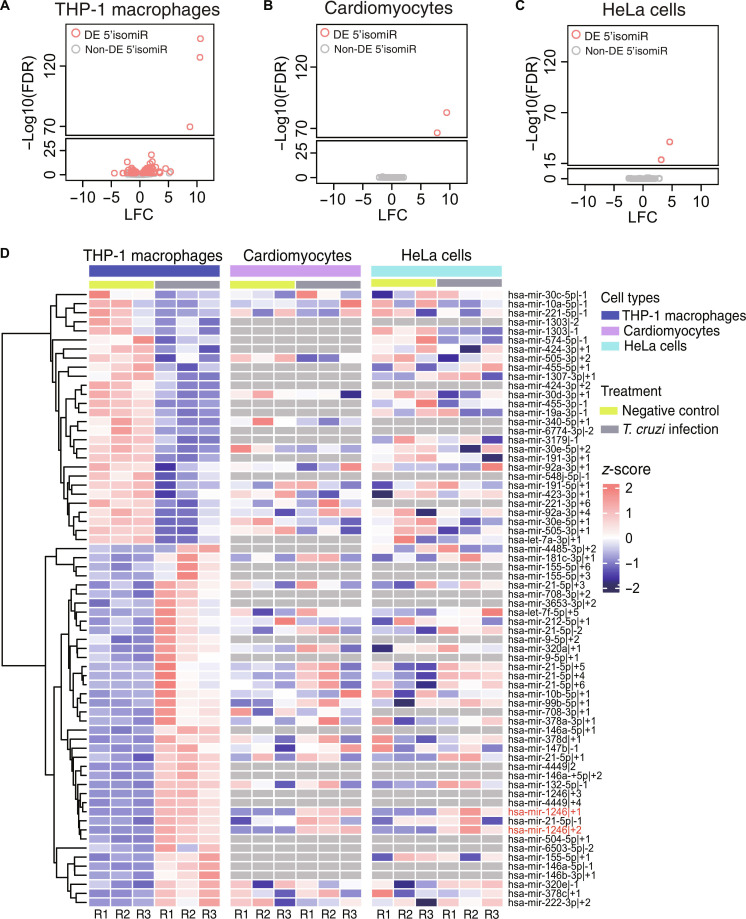
*Trypanosoma cruzi* infection selectively reprograms 5’ isomiR expression in macrophages. (A to C) Volcano plots of differentially expressed (DE) 5’ isomiR (in salmon, DE 5’ isomiR) at 24 h post *Trypanosoma cruzi* infection in THP-1-derived macrophages (A), primary cardiomyocytes (B), and HeLa cells (C). The 5’ isomiR without differential expression were in gray (Non-DE 5’ isomiR). (D) Heatmap showing the expression abundance comparison of 68 DE 5’ isomiR at 24 h post *Trypanosoma cruzi* infection in THP-1-derived macrophages, primary cardiomyocytes, and HeLa cells.

We next sought to determine whether the increased 5’-end heterogeneity observed in *T. cruzi-*infected macrophages is accompanied by changes in the expression levels of specific 5’ isomiRs. We identified 499 5’ isomiRs in *T. cruzi-*infected macrophages (Table S1). The principal component analysis showed clear separation between the infected group and the negative control groups (Fig. S1). Differential expression analysis using edgeR identified 68 significantly dysregulated 5’ isomiRs in THP-1-derived macrophages post *T. cruzi* infection, and 65 out of 68 5’ isomiRs were also supported by DESeq2 (negative binomial test, FDR < 0.05; Fig. 2A, Fig. S2, and Table S1), indicating substantial reprogramming of the 5’ isomiRs landscape in *T. cruzi-*infected macrophages. In contrast, only 2 differentially expressed 5’ isomiRs were detected in both primary cardiomyocytes and epithelial cells (Fig. 2B and C and Tables S2 and S3). Consistent with the volcano plot results, the heatmap of the 68 dysregulated 5’ isomiRs demonstrated a strong cell-type specificity of 5’ isomiR regulation during *T. cruzi* infection (Fig. 2D). Among the top 5 dysregulated 5’ isomiRs in *T. cruzi-*infected macrophages, 3 were derived from the miR-1246 precursor, including miR-1246|+1, miR-1246|+2, and miR-1246|+3. These 3 isoforms displayed strongest induction and high expression abundance post infection in macrophages (Fig. 2A). Further examination of the raw read count distribution confirmed that the elevated expression of these 3 isoforms was consistently observed across replicates rather than being driven by a single highly expressed sample (Table S1). Although miR-1246|+1 and miR-1246|+2 were also significantly up-regulated after *T. cruzi* infection in primary cardiomyocytes and epithelial cells, their induction and abundance were relatively lower (Fig. 2B and C). Together, these results show strong cell-type specificity in 5’ isomiR reprogramming following *T. cruzi* infection, with infected macrophages displaying substantially greater and more selective 5’ isomiR dysregulation than primary cardiomyocytes or epithelial cells.

### Most dysregulated 5’ isomiRs are specific to *T. cruzi* infection in macrophages

**Fig. 3.**
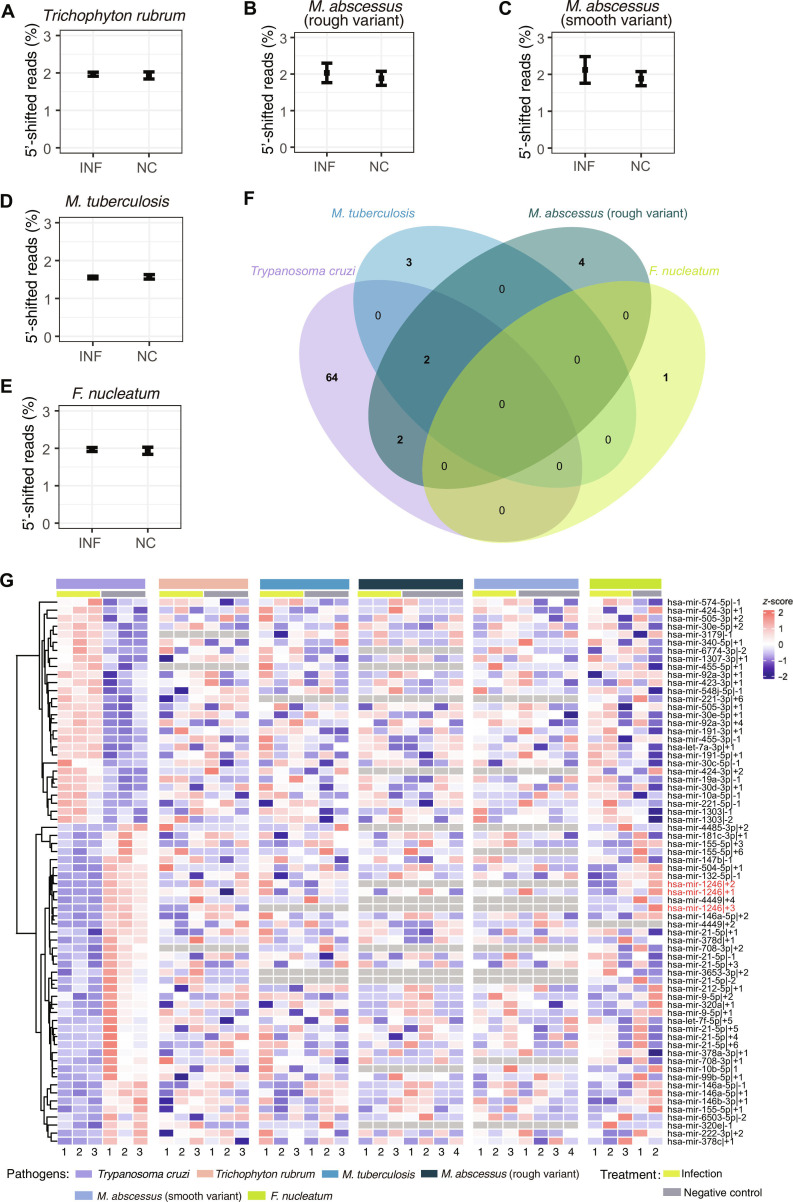
Most dysregulated 5’ isomiRs are specific to *Trypanosoma cruzi* infection in macrophages. (A to E) Proportions of reads exhibiting 5’-end shifts to the upstream or downstream of cognate anomiRs in THP-1-derived macrophages infected with *Trichophyton rubrum* (A), *M. abscessus* (rough variant) (B), *M. abscessus* (smooth variant) (C), *M. tuberculosis* (D), and *F. nucleatum* (E). (F) Venn diagram of differentially expressed 5’ isomiRs in THP-1-derived macrophages infected with *Trichophyton rubrum*, *M. tuberculosis*, *M. abscessus* (rough variant), and *F. nucleatum*. (G) For 68 dysregulated 5’ isomiRs identified in *Trypanosoma cruzi*-infected macrophages, heatmap showing the expression abundance comparison in THP-1-derived macrophages infected with *Trichophyton rubrum*, *M. tuberculosis*, *M. abscessus* (rough variant), *M. abscessus* (smooth variant), and *F. nucleatum*.

To determine whether the observed 5’ isomiR reprogramming represents a general response to microbial infection or a *T. cruzi*-specific phenomenon, we compared 5’ isomiR profiles across macrophages infected with multiple pathogens (Materials and Methods). Analysis of 5’-end shift distributions revealed that infections with *Trichophyton rubrum*, *Mycobacterium abscessus* (rough and smooth variants), *Mycobacterium tuberculosis*, and *Fusobacterium nucleatum* did not induce comparable increases in 5’-end heterogeneity (Fig. 3A to E), indicating that the pronounced heterogeneity observed in *T. cruzi*-infected macrophages is not a universal feature of infection. We next performed overlap analysis of differentially expressed 5’ isomiRs across these pathogen-infected macrophages. Notably, comparative expression profiling showed that 64 out of the 68 dysregulated 5’ isomiRs identified in *T. cruzi*-infected macrophages were either undetectable or not differentially expressed in macrophages infected with other pathogens (Fig. 3F and G and Tables S4 to S8), demonstrating a high degree of pathogen specificity. Collectively**,** these results indicated that 5’ isomiR remodeling in macrophages represented a highly specific response to *T. cruzi* infection, rather than a general consequence of microbial stimulation or inflammatory activation.

### Dysregulated 5’ isomiRs are predominantly loaded onto AGO proteins in macrophages

**Fig. 4.**
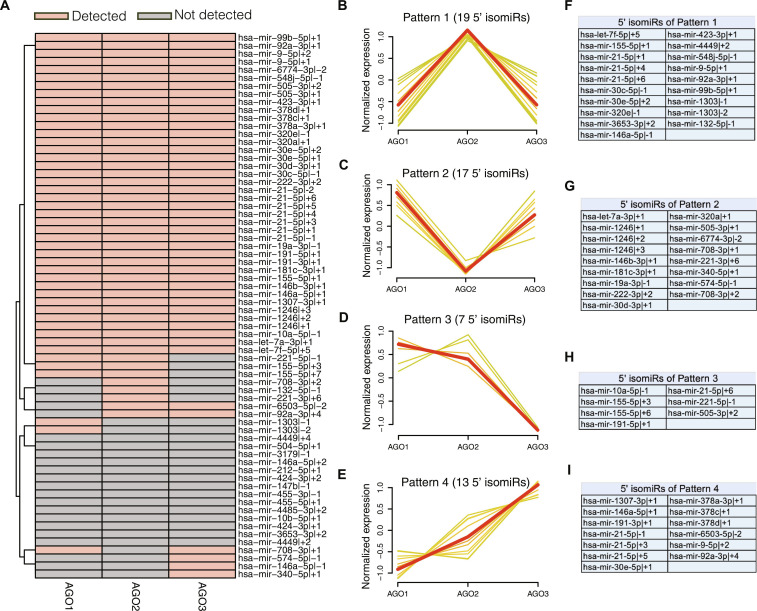
Dysregulated 5’ isomiRs are predominantly loaded onto Argonaute proteins in macrophages. (A) Summary of the 5’ isomiRs detection across 3 Argonaute proteins (AGO1, AGO2, and AGO3) for the 68 dysregulated 5’ isomiRs identified in *Trypanosoma cruzi*-infected THP-1 macrophage. (B to E) Expression pattern of 56 5’ isomiRs that detected in at least 1 Argonaute protein and exhibited expression difference among 3 Argonaute protein in THP-1 cells. (F to I) 5’ IsomiR members of clusters of Pattern 1, 2, 3, and 4, respectively.

To assess the functional relevance of the dysregulated 5’ isomiRs identified in *T. cruzi-*infected macrophages, we examined their association with AGO proteins in THP-1 cells, which are essential components of the RISC [39,40]. Among the 68 differentially expressed 5’ isomiRs, 56 (82.4%) were detected in at least one of the AGO1, AGO2, or AGO3 immunoprecipitation small RNA-seq datasets (Fig. 4A), indicating that the majority of these dysregulated 5’ isomiRs were actively incorporated into the miRNA silencing machinery. Further clustering analysis revealed distinct patterns of AGO association among these 5’ isomiRs (Fig. 4B to I and Table S9), suggesting that individual 5’ isomiR may exhibit preferential loading into specific AGO proteins. This selective loading pattern also suggested an additional level of regulation, whereby distinct 5’ isomiRs may differentially contribute to gene silencing. Together, these findings demonstrated that *T. cruzi* infection 5’ isomiRs were not by-products of imprecise miRNA processing but may actively engage in posttranscriptional regulation.

### miR-1246|+1 represses transcriptional programs of target genes associated with NF-κB signaling, cytokine responses, and cell migration

**Fig. 5.**
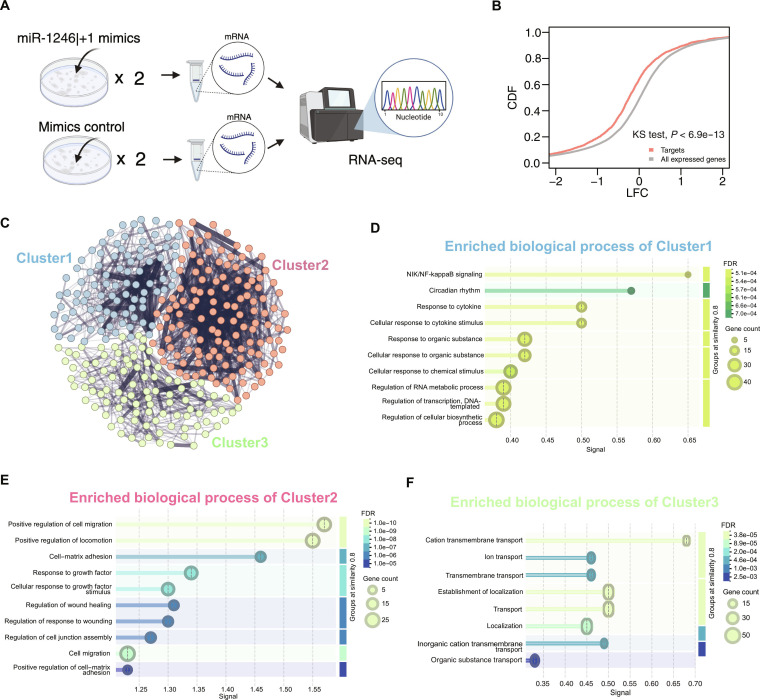
miR-1246|+1 represses transcriptional programs of target genes associated with nuclear factor-κB (NF-κB) signaling, cytokine responses, and cell migration. (A) Scheme of the experiment design for measuring the regulatory effects of miR-1246|+1 mimics compared with mimics control using RNA sequencing (RNA-seq) profiling. The experiments were conducted in 2 biological replicates. (B) The cumulative distribution function (CDF) of the expression changes of predicted target genes of miR-1246|+1 and all expressed genes (Background) after miR-1246|+1 mimics transfection. (C) Clusters of protein–protein interaction (PPI) network of significantly down-regulated miR-1246|+1 target genes. (D to F) Significantly enriched biological processes of miR-1246|+1 target genes of Cluster1 (D), Cluster2 (E), and Cluster3 (F). In (D) to (F), the signal is defined as a weighted harmonic mean of the observed/expected ratio and −log_10_(FDR), balancing enrichment magnitude and statistical significance for intuitive ranking of enriched terms. The enriched items were grouped under the similarity 0.8. KS, Kolmogorov–Smirnov test.

Given the prominent induction and AGO association of miR-1246-derived 5’ isomiRs, we focused on miR-1246|+1, a 5’ isomiR exhibiting a 1-nucleotide downstream shift at the 5’ end of canonical miR-1246, for further functional characterization. To evaluate its regulatory impact, THP-1-derived macrophages were transfected with miR-1246|+1 mimics, followed by transcriptome profiling using RNA-seq (Fig. 5A and Materials and Methods). Cumulative distribution analysis revealed that the overall mRNA abundance of predicted target genes of miR-1246|+1 were significantly down-regulated compared with all expressed genes post miR-1246|+1 mimics transfection (Kolmogorov–Smirnov test, *P* value < 6.9 × 10^−13^; Fig. 5B), resulting a total of 321 significantly repressed target genes using edgeR (negative binomial test, FDR < 0.05; Tables S10 and S11). The majority of these repressed target genes were also supported by DESeq2 (Fig. S3 and Table S10). We further performed network analysis to analyze these significantly repressed target genes in more detail. PPI network construction and clustering analysis classified these significantly down-regulated target genes into 3 network clusters (Fig. 5C, Figs. S4 to S6, and Table S11). Functional enrichment analysis revealed distinct function of each cluster. Specifically, Cluster1 (number of nodes: 97; observed number of edges: 522; expected number of edges: 262; PPI enrichment *P* value: 1.0 × 10^−13^) were strongly associated with biological processes related to NF-κB signaling, circadian rhythm, and cytokine-mediated immune responses (Fig. 5D and Table S12). Cluster2 (number of nodes: 116; observed number of edges: 939; expected number of edges: 379; PPI enrichment *P* value: 2.6 × 10^−17^) were significantly enriched in cell migration related processes (Fig. 5E and Table S12). Cluster3 (number of nodes: 108; observed number of edges: 389; expected number of edges: 208; PPI enrichment *P* value: 1.0 × 10^−8^) were linked with the processes related to cation transmembrane transport and ion transport (Fig. 5F and Table S12). Notably, most of these enriched biological processes and pathways are central to macrophage activation, trafficking, polarization, and inflammatory responses [9,12,14,41–43], indicating that miR-1246|+1 may serve as a broad regulator of immune-related transcriptional programs in macrophages.

To further explore the relevance of the enrichment of circadian rhythm, we examined the 7 genes contributing to this process in Cluster1 (Bhlhe41, Arntl2, Egr3, Klf10, Slc6a4, Nampt, and Tnfrsf11a). These genes did not form an independent interaction module within the PPI network of Cluster1 (Fig. S4). We next assessed whether these genes exhibited rhythmic expression in macrophages using publicly available circadian transcriptomic data from murine peritoneal CD11b^+^ macrophages [38] (Materials and Methods). Among the 7 genes, Bhlhe41 displayed significant rhythmic expression, whereas the remaining genes showed limited or no evidence of rhythmicity (Fig. S7). Notably, Bhlhe41 has recently been identified as a marker of a reparative macrophage population involved in limiting fibrosis and promoting cardiac remodeling following myocardial infarction [44], and its dysregulation might be related to chronic inflammatory cardiomyopathies such as those observed in Chagas disease. Together, these findings suggest that the circadian rhythm enrichment observed in Cluster1 is linked to macrophage inflammatory and functional programs rather than representing an isolated circadian transcriptional module.

### miR-1246|+1 attenuates NF-κB-driven inflammatory responses and restricts M1 macrophage phenotype

**Fig. 6.**
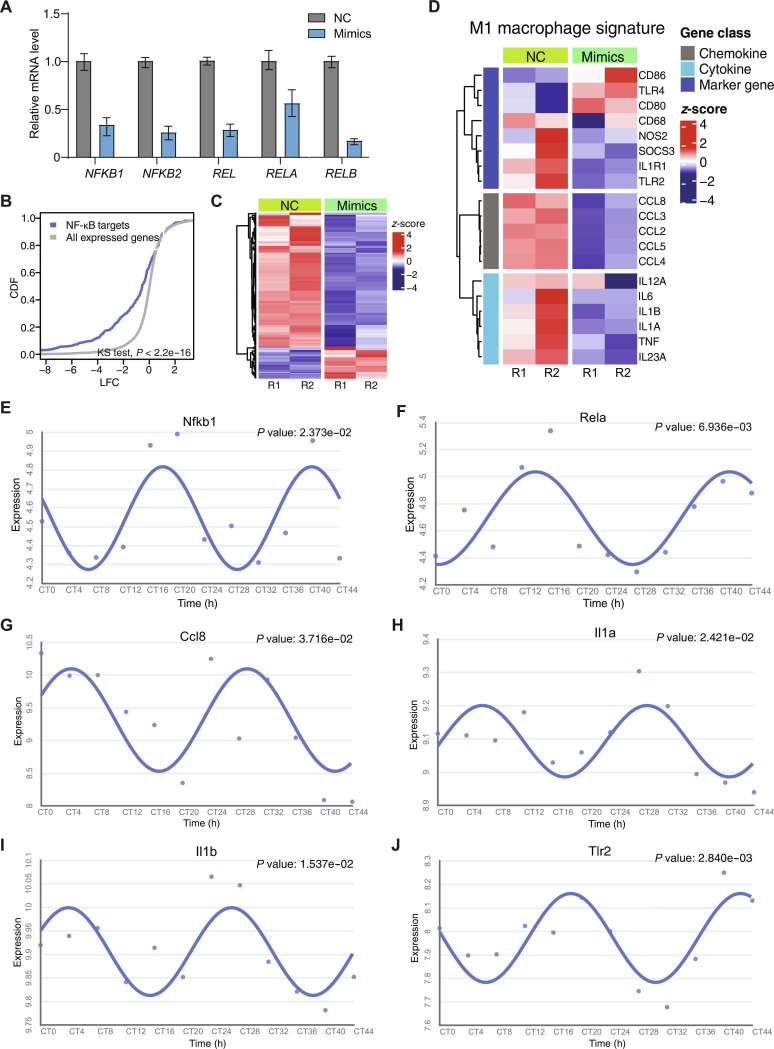
miR-1246|+1 attenuates nuclear factor-κB (NF-κB)-driven inflammatory responses and restricts M1 macrophage phenotype. (A) Quantitative polymerase chain reaction (qPCR) result of the mRNA expression levels of NF-κB transcription factors after miRNA-1246|+1 mimics transfection (Mimics). Mimics control transfection was used as negative control (NC) (*n* = 5). (B) The cumulative distribution function (CDF) of the expression changes of target genes of NF-κB transcription factors and all expressed genes (Background) after miR-1246|+1 mimics transfection. (C) Heatmap showing the expression changes of significantly dysregulated target genes of NF-κB transcription factors after miRNA-1246|+1 mimics transfection. (D) Heatmap showing the expression changes of established marker genes, chemokine, and cytokine of M1 macrophage after miRNA-1246|+1 mimics transfection. (E to J) Two NF-κB family transcription factors, Nfkb1 (E) and Rela (F), as well as 4 M1 macrophage signature genes, including Ccl8 (G), Il1a (H), Il1b (I), and Tlr2 (J), exhibited rhythmic expression pattern in mouse peritoneal macrophages. KS, Kolmogorov–Smirnov test.

NF-κB signaling is a central regulator of inflammatory macrophage activation and is closely linked to the induction of antimicrobial and proinflammatory effector programs that contribute to resistance against *T. cruzi* infection and shape the M1-like phenotype [9,12,14,41,42]. To further investigate the functional consequences of miR-1246|+1-mediated regulation, we examined its effects on NF-κB signaling and macrophage polarization. Quantitative PCR analysis demonstrated that overexpression of miR-1246|+1 significantly reduced the mRNA levels of multiple NF-κB transcription factors, including *NFKB1*, *NFKB2*, *REL*, *RELA*, and *RELB* (Fig. 6A). Consistent with these results, cumulative distribution analysis showed that genes regulated by NF-κB transcription factors were globally down-regulated following miR-1246|+1 overexpression (Kolmogorov–Smirnov test, *P* value < 2.2 × 10^−16^; Fig. 6B). Moreover, 118 out of 146 differentially expressed NF-κB target genes after miR-1246|+1 overexpression was deceased (Fig. 6C). Importantly, M1 macrophage signature analysis showed that miR-1246|+1 also led to a marked reduction in the expression of most of established M1 macrophage markers, as well as proinflammatory cytokines and chemokines (Fig. 6D), indicating suppression of classical inflammatory macrophage activation. Interestingly, extending the analysis to examine circadian rhythmicity revealed that, in addition to Cluster1 gene Bhlhe41, several NF-κB family transcription factors (Nfkb1 and Rela) as well as canonical M1 macrophage signature genes (Ccl8, Il1a, Il1b, and Tlr2) also exhibited rhythmic expression in murine peritoneal macrophages (Fig. 6E to J), suggesting a potential convergence between circadian-associated transcriptional regulation and NF-κB-driven inflammatory programs. Taken together, these findings demonstrated that miR-1246|+1 may function as a potent negative regulator of NF-κB-driven inflammatory responses and M1 macrophage phenotype, providing a mechanistic link between 5’ isomiR remodeling and immune modulation during *T. cruzi* infection.

## Discussion

This study identified 5’ isomiR remodeling as a previously underappreciated regulatory layer in host–pathogen interactions and provided evidence that *T. cruzi* infection induced a highly selective rewiring of miRNA seed region in macrophages. Unlike conventional analyses focused on total miRNA abundance, our results highlighted the importance of isoform-level variation, particularly at the 5’ end, which directly determines target specificity and thereby expands the functional scope of the miRNA regulatory network. miRNAs are well-established regulators of macrophage activation and inflammatory signaling, especially through modulation of NF-κB pathways [9,12,14,41–43]. However, most studies have implicitly treated miRNAs as single functional entities. Our findings extend this paradigm by demonstrating that infection can reshape miRNA function at the level of seed sequence identity through selective induction of 5’ isomiRs. This observation is in line with emerging evidence that isomiRs contribute to regulatory diversity and can exhibit distinct targeting properties compared with their canonical counterparts [20,21].

Recent studies have emphasized the potential of computational approaches to address *T. cruzi* infection and protozoan diseases more broadly. Ros-Lucas et al. [45] applied bioinformatics-guided virtual screening using AlphaFold-predicted *T. cruzi* protein structures to identify novel compounds, demonstrating the feasibility of in silico drug repurposing against previously uncharacterized parasitic targets. Similarly, Hashim and Dimier-Poisson [46] reviewed computational vaccinology strategies for protozoan parasites, highlighting immuno-informatics and reverse vaccinology approaches to identify conserved epitopes and design multivalent vaccines. These works collectively underscore the value of integrating computational and experimental analyses to accelerate discovery and mechanistic understanding in protozoan infection contexts. Our integrative computational study adds another layer of posttranscriptional regulation following *T. cruzi* infection in macrophage. A notable finding of our results is the strong cell-type specificity of 5’ isomiR remodeling. Macrophages, but not cardiomyocytes or epithelial cells, exhibited substantial increases in 5’ heterogeneity and 5’ isomiR reprogramming. This specificity likely reflects the unique immunological role of macrophages as both effector cells and intracellular reservoirs during *T. cruzi* infection [8,11,47]. It also raises the possibility that miRNA biogenesis or stability is dynamically regulated in a lineage-dependent manner, potentially through modulation of Drosha/Dicer processing or AGO loading under inflammatory conditions [39,48], which requires further exploration.

Equally notable is the pathogen specificity of the observed response. The finding that 64 out of 68 dysregulated 5’ isomiRs are unique to *T. cruzi* infection argued against a generic stress- or inflammation-driven mechanism and instead supported a model of pathogen-tailored regulatory remodeling. This is consistent with previous studies showing that *T. cruzi* actively suppresses macrophage inflammatory responses, including NF-κB signaling, to promote intracellular survival [10,12]. Our data suggested that such immune modulation may extend to the posttranscriptional level through 5’ isomiR-mediated rewiring of gene regulatory networks.

Functional miRNAs are typically loaded into AGO proteins to form RISCs, where AGO association is a key determinant of miRNA stability and silencing activity [39,40]. The functional relevance of dysregulated 5’ isomiRs identified in this study is supported by their widespread association with AGO proteins. The majority of dysregulated 5’ isomiRs were detected in AGO complexes, indicating their active participation in RNA silencing. This observation reinforces the notion that isomiRs are not merely by-products of imprecise processing but are selectively stabilized and functionally engaged regulatory molecules [20]. Notably, previous studies have shown that human AGO proteins associate with distinct pools of small RNAs and may contribute to isoform-specific sorting and silencing output [40,49], which is in line with the distinct AGO-loading patterns observed in this study. Among the identified 5’ isomiRs, miR-1246|+1 emerged as a key regulator. Our results demonstrated that miR-1246|+1 broadly suppressed NF-κB transcription factors and downstream inflammatory genes, resulting in inhibition of M1 macrophage responses. Given the central role of NF-κB in orchestrating antimicrobial responses and immune activation [9,12,14,41–43], this regulatory effect may likely to have regulatory consequences for host defense and pathogen persistence.

Our analysis suggests a potential link between circadian-associated transcriptional programs and macrophage responses to *T. cruzi* infection. Macrophages possess intrinsic circadian clocks that regulate inflammatory immune outputs. Keller et al. demonstrated that murine peritoneal macrophages contain autonomous circadian clockworks and that circadian timing regulates inflammatory cytokine responses, including tumor necrosis factor-α and interleukin-6 (IL-6) production [38]. Consistent with this concept, our re-analysis of this microarray dataset revealed rhythmic expression of Bhlhe41 in mouse peritoneal macrophages, as well as rhythmic expression of NF-κB family members and M1-associated inflammatory genes. These findings suggest that the “circadian rhythm” enrichment observed in Cluster1 does not simply reflect canonical timekeeping machinery but may instead represent a broader macrophage regulatory program coupled to inflammatory activation. Bhlhe41 may represent a particularly important node within this program. Bhlhe40/Bhlhe41 transcription factors have been shown to regulate tissue-resident macrophage identity, self-renewal, and tissue adaptation, including in alveolar macrophages [50]. Moreover, a recent study identified Bhlhe41+ macrophages as a reparative macrophage population in the infarcted heart that limits excessive fibrosis and promotes remodeling of the developing infarcted myocardium [44]. In the present study, we found that miR-1246|+1 is robustly induced by *T. cruzi* infection and that Bhlhe41 is a predicted and experimentally validated target of miR-1246|+1. Thus, infection-induced miR-1246|+1 may suppress a Bhlhe41-centered macrophage program that integrates circadian-associated regulation with tissue-protective immune functions. Given that *T. cruzi* infection is the etiological agent of Chagas disease and can lead to chronic inflammatory cardiomyopathy, heart failure, arrhythmias, and other severe cardiac complications [4–6], our findings raise the possibility that the inhibition of Bhlhe41 following miR-1246|+1 overexpression may contribute to maladaptive macrophage activation and cardiac pathology during infection. Together, these results identify Bhlhe41 as a potential molecular bridge connecting circadian-regulated macrophage states, parasite-induced miRNA remodeling, and inflammatory cardiac disease. Future studies defining how temporal macrophage programs are rewired during *T. cruzi* infection may provide new insight into the immunometabolic and transcriptional mechanisms underlying Chagas cardiomyopathy.

Our findings should be viewed within the broader context of macrophage regulatory mechanisms that have been described during *T. cruzi* infection. Previous studies demonstrated that immunometabolic reprogramming contributes to macrophage activation by promoting oxidative metabolism required for nitric oxide production and antiparasitic responses [51]. In addition, posttranscriptional regulation of inflammatory effector molecules has been reported, as *T. cruzi*-induced nitric oxide production is influenced by changes in inducible nitric oxide synthase mRNA stability [52]. Receptor-mediated signaling pathways also modulate macrophage polarization during infection. For example, Axl-dependent efferocytosis suppresses M1 macrophage responses and promotes parasite persistence [12], whereas inhibition of mechanistic target of rapamycin signaling alters macrophage intracellular programs and affects infection outcomes [53]. Together, these studies indicate that macrophage inflammatory responses to *T. cruzi* are regulated through multiple interconnected mechanisms, including metabolic adaptation, signaling pathway modulation, and posttranscriptional control. Our work identifies 5′ isomiR remodeling as an additional regulatory layer that may cooperate with these established pathways to fine-tune macrophage activation during infection.

Interferon-γ (IFN-γ) is one of the most important cytokines for protective immunity against *T. cruzi* infection. IFN-γ produced by natural killer cells and T cells promotes classical macrophage activation, nitric oxide production, and parasite control, and defects in IFN-γ-associated responses can impair resistance to infection [54]. In the present study, specific IFN-γ expression assays were not performed because the experimental system focused on THP-1-derived macrophages cultured in isolation, whereas IFN-γ is primarily produced by lymphoid cells rather than macrophages. Nevertheless, miR-1246|+1 mediated suppression of NF-κB, and M1-associated transcriptional programs may influence how macrophages respond to IFN-γ in a multicellular immune environment. In addition to T helper 1 (Th1) immunity, Th17-associated pathways have also been implicated in *T. cruzi* infection. IL-17 has been reported to participate in host defense, modulate myocarditis, and correlate with clinical outcomes in Chagas disease [55]. These observations indicate that parasite control and cardiac pathology are shaped by a complex cytokine network involving Th1, Th17, regulatory, and macrophage-derived inflammatory pathways. Future studies using macrophage–lymphocyte coculture systems, primary immune cells, and *in vivo* Chagas disease models will be important to determine whether miR-1246|+1-mediated 5′ isomiR remodeling affects IFN-γ responsiveness, Th17-associated inflammation, and macrophage–lymphocyte crosstalk during *T. cruzi* infection.

Although cardiomyocytes are not professional immune cells, they actively contribute to the inflammatory and pathological environment of Chagas disease. *T. cruzi*-infected cardiomyocytes can activate innate immune signaling, produce cytokines and chemokines, undergo oxidative and mitochondrial stress, and contribute to leukocyte recruitment and myocardial injury [56]. In our analysis, cardiomyocytes exhibited far fewer differentially expressed 5′ isomiRs than THP-1-derived macrophages. This difference does not imply that cardiomyocytes are immunologically inert; rather, it suggests that 5′ isomiR remodeling may be a more prominent feature of professional immune cells during *T. cruzi* infection. Macrophages function as both antimicrobial effector cells and intracellular host cells for *T. cruzi* and therefore may require broader posttranscriptional rewiring to coordinate inflammatory activation, parasite control, and immune modulation. By contrast, cardiomyocyte responses may be more restricted or specialized, involving stress-response pathways, innate immune sensing, chemokine production, and tissue-injury programs rather than extensive isomiR remodeling. This cell-type specificity is consistent with the concept that miRNA and isomiR responses to infection are shaped by cellular identity and immune function.

This study has several limitations. (a) Among the 68 *T. cruzi*-responsive 5′ isomiRs, 56 were detected in at least 1 AGO1-, AGO2-, or AGO3-associated small RNA dataset from THP-1 cells, supporting their potential functional engagement with the miRNA silencing machinery. Because these AGO datasets were not generated under *T. cruzi* infection, they indicate AGO association in a THP-1 cellular context but do not directly establish infection-specific AGO loading. The remaining 12 isomiRs are considered lower-confidence functional candidates that require additional validation. (b) Although DESeq2 reanalysis, raw count inspection, principal component analysis, and the miR-1246|+1 mimic RNA-seq dataset collectively supported the robustness of the identified 5′ isomiRs and their target genes, the limited number of biological replicates may still affect statistical power and false-positive control. Further validation in larger independent cohorts will be important to confirm these findings. (c) THP-1-derived macrophages provide a reproducible model for studying *T. cruzi* infection, but they do not fully recapitulate the biology of primary human macrophages or the phenotypes of cardiac-resident macrophages. THP-1 cells are derived from a monocytic leukemia line and require PMA-induced differentiation, which may alter basal transcriptional and inflammatory states, influence NF-κB responsiveness, and potentiate cytokine production independent of infection. Resident cardiac macrophages, by contrast, are shaped by ontogeny, local tissue signals, cell–cell interactions, metabolic cues, and inflammatory microenvironments, conditions that are not reproduced *in vitro*. Consequently, the regulatory effects of miR-1246|+1 observed in THP-1 cells should be validated in primary monocyte-derived macrophages, tissue-resident macrophage models, and *in vivo* infection systems to determine how epigenetic, translational, and metabolic mechanisms interact during *T. cruzi* infection. (d) Although our transcriptomic analyses indicate that miR-1246|+1 suppresses NF-κB-dependent transcription and M1-like inflammatory signatures, these results do not directly measure cytokine secretion or effector activity. NF-κB regulates multiple cytokines including tumor necrosis factor-α, IL-1β, IL-6, and IL-12. In *T. cruzi* infection, these cytokines are essential for macrophage-mediated parasite control, while dysregulated production contributes to tissue pathology. Thus, down-regulation of NF-κB targets suggests likely attenuation of inflammatory function, but protein-level studies (enzyme-linked immunosorbent assay, multiplex assays, and flow cytometry) are required for confirmation.

## Conclusion

In summary, this study demonstrates that *T. cruzi* infection induces a highly selective and functionally relevant remodeling of the 5’ isomiR landscape in macrophages. This remodeling is both cell type- and pathogen-specific and is characterized by the preferential generation and AGO loading of 5’ isomiRs. We identify miR-1246|+1 as a potencial regulator that suppresses NF-κB signaling and attenuates inflammatory macrophage activation, thereby linking 5’ isomiR remodeling to immune modulation. These findings establish 5’ isomiR rewiring as a novel regulatory mechanism in host–pathogen interactions and highlight 5’ isomiRs as potential targets for therapeutic intervention.

## Data Availability

All original RNA-Seq data have been deposited in GEO database with accession number GSE328447. The code for data analysis has been deposited in Zenodo following the link: https://zenodo.org/records/20438193.
