# Supplementary material for: Comparative 5’ IsomiRome Analysis Uncovers Dysregulated 5’ IsomiRs in Trypanosoma cruzi-infected Macrophages
Source: Comput Struct Biotechnol J. 2026 Jun 24;35(1):0146. doi: 10.34133/csbj.0146 (PMC13484906; doi:10.34133/csbj.0146)
Supplement: Supplementary file 1 [file csbj.0146.f1.zip › Supplementary Figures.pdf]

Supplementary Figures

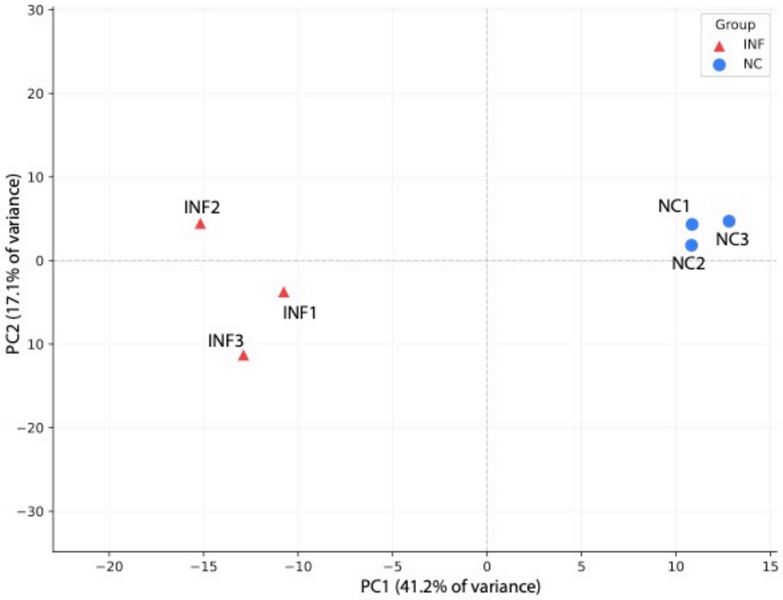

Figure S1. PCA plot of identified 5'isomiRs in Trypanosoma cruzi-infected THP-1 derived macrophages. Trypanosoma cruzi-infected samples were abbreviated as INF1-3, and corresponding negative control samples were abbreviated as NC1-3.

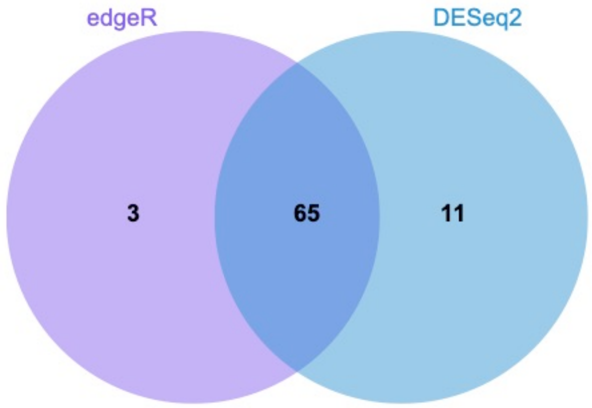

Figure S2. Overlaps of differentially expressed 5'isomiRs between edgeR and DESeq2. The differentially expressed 5'isomiRs were identified using edgeR or DESeq2 with the criteria of FDR < 0.05 and FC>1.5.

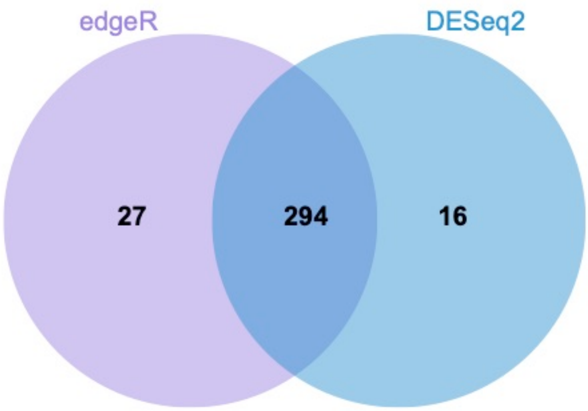

Figure S3. Overlaps of differentially expressed miR-1246|+1 target genes between edgeR and DESeq2. The differentially expressed miR-1246|+1 target genes were identified using edgeR or DESeq2 with the criteria of FDR < 0.05 and FC>1.5.

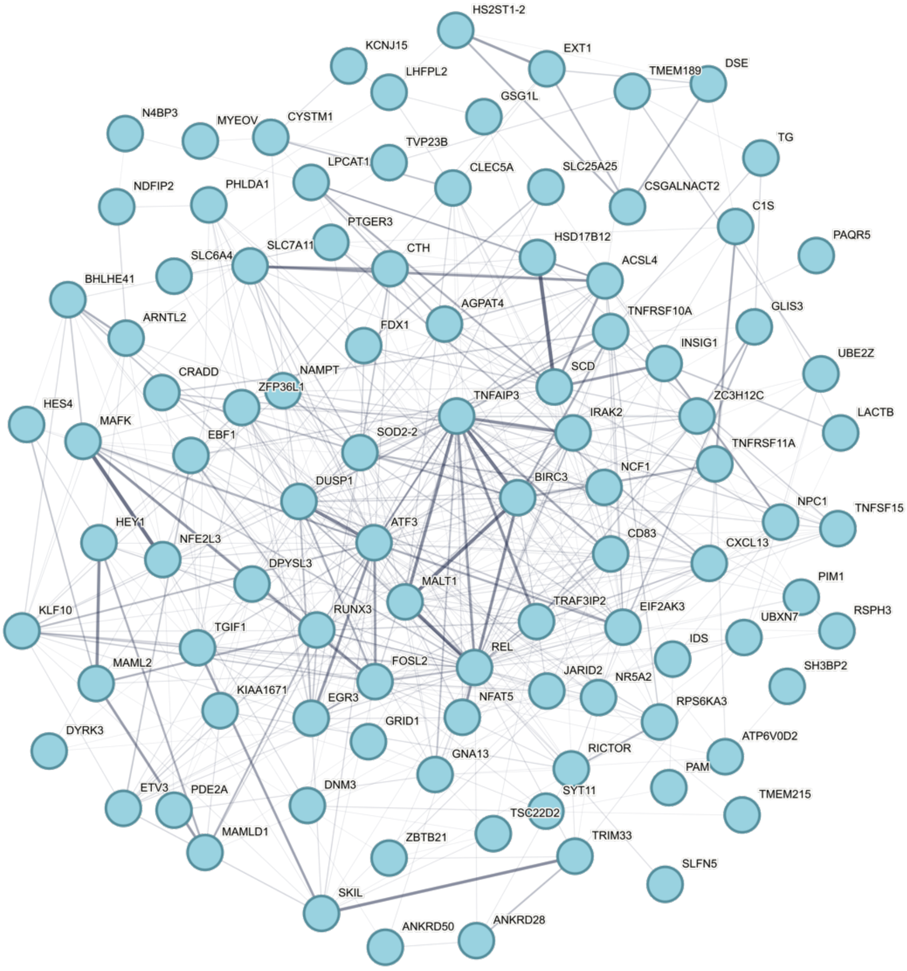

Figure S4. Protein-protein interaction of Cluster1 genes. The Protein-protein interaction information was obtained from STRING database. Each blue node represents a cluster1 gene. The edge thickness is proportional to the interaction confidence score.

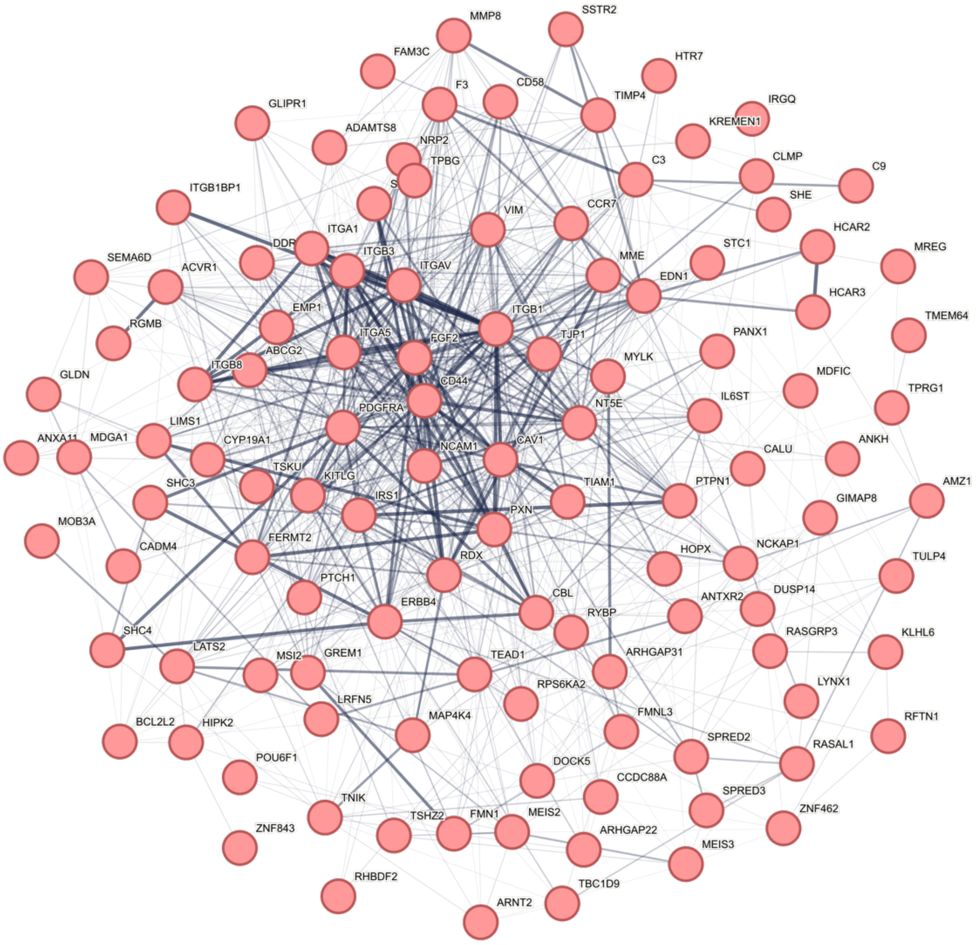

Figure S5. Protein-protein interaction of Cluster2 genes. The Protein-protein interaction information was obtained from STRING database. Each salmon node represents a cluster2 gene. The edge thickness is proportional to the interaction confidence score.

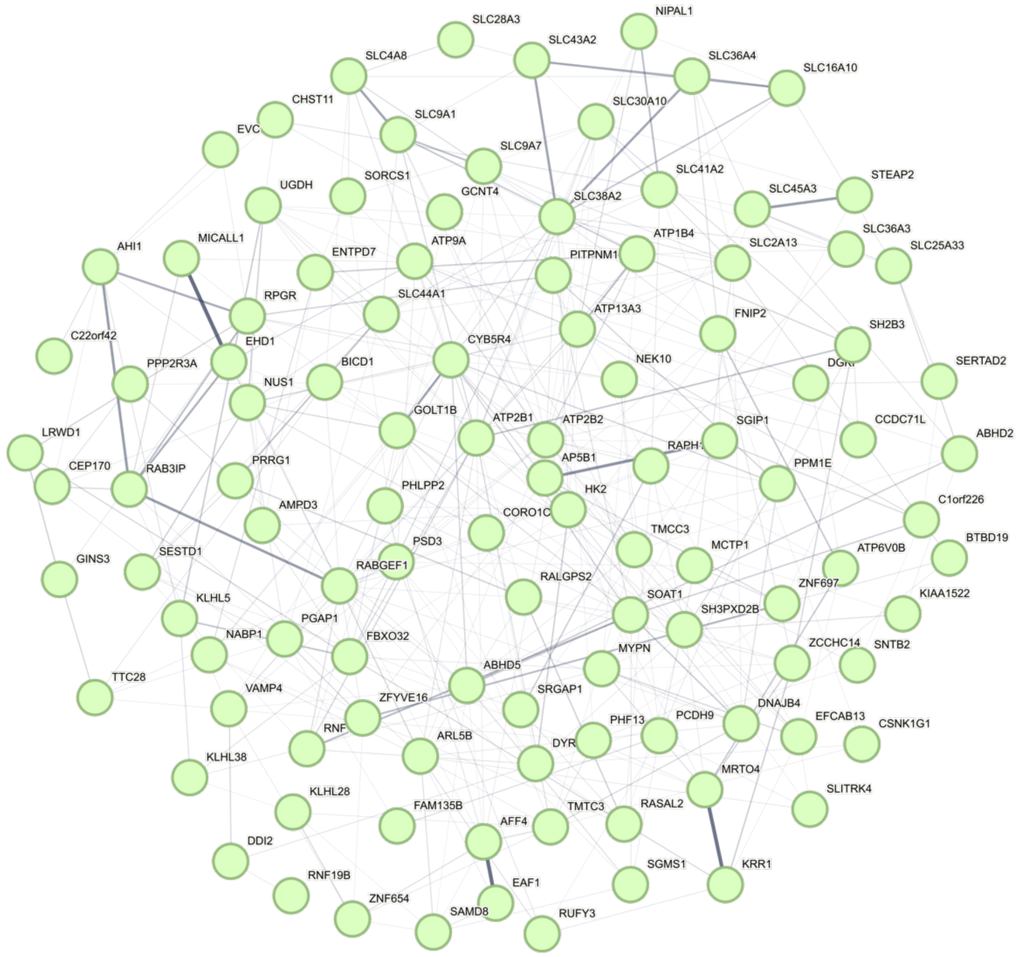

Figure S6. Protein-protein interaction of Cluster3 genes. The Protein-protein interaction information was obtained from STRING database. Each green node represents a cluster3 gene. The edge thickness is proportional to the interaction confidence score.

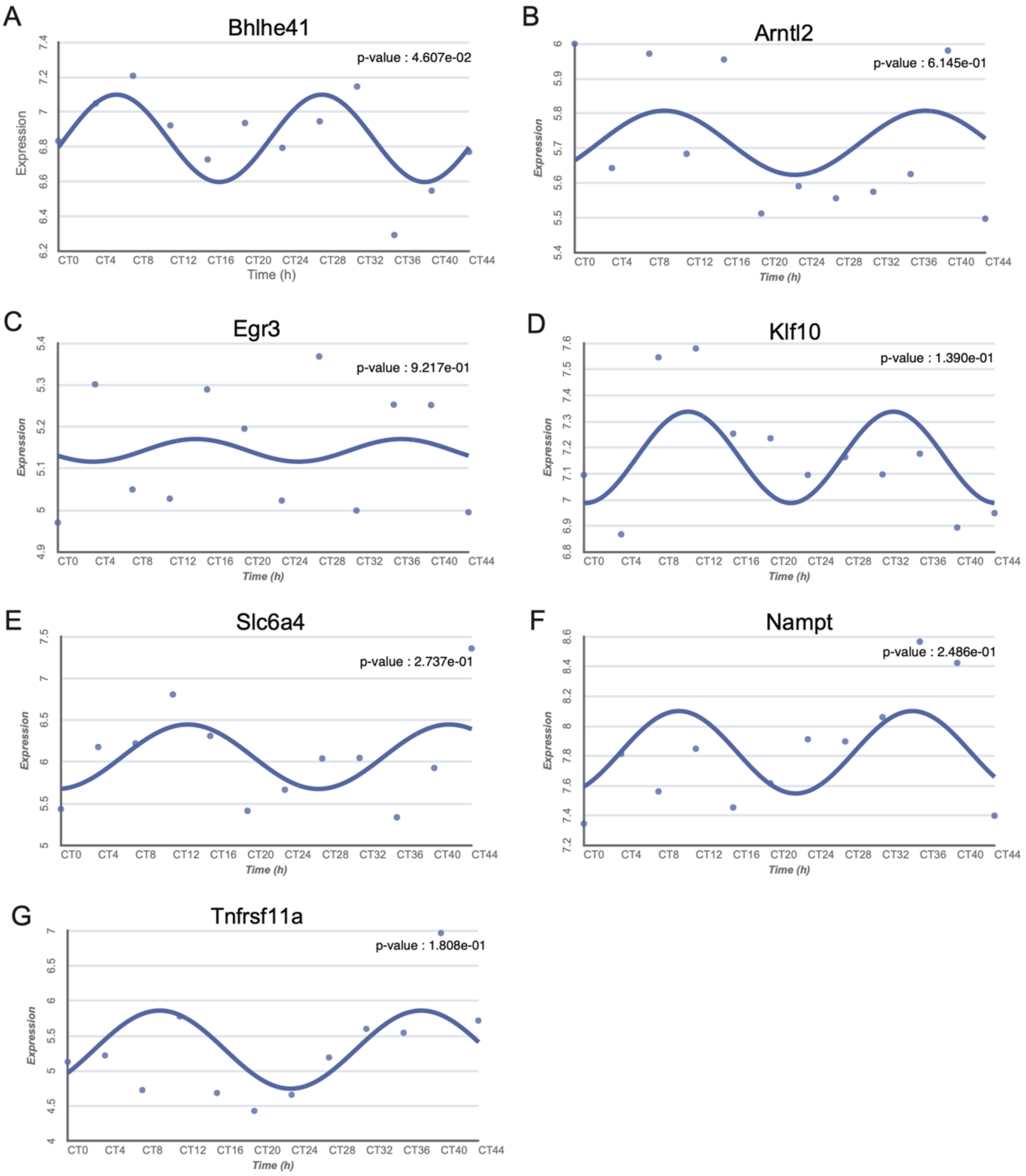

Figure S7. Bhlhe41 is rhythmically expressed in mouse peritoneal macrophages. Mice were entrained to a 12-h light/12-h dark (LD) cycle for 2 weeks and then transferred to constant conditions, specifically constant darkness (DD) with water and food available *ad libitum*. Peritoneal CD11b+ macrophages were collected every 4 h over 48 h, and gene expression was profiled by microarray, as reported by Keller et al. (2009, *PNAS*). We used this microarray dataset from CircaKB to determine whether seven cluster 1 genes (*Bhlhe41*, *Arntl2*, *Egr3*, *Klf10*, *Slc6a4*, *Nampt*, and *Tnfrsf11a*) annotated as being involved in the circadian rhythm biological process, exhibit circadian rhythmic expression in mouse peritoneal macrophages.
